# Valence Electronic Structure of Interfacial Phenol in Water Droplets

**DOI:** 10.1021/acs.jpca.4c04269

**Published:** 2024-08-25

**Authors:** Jonas Heitland, Jong Chan Lee, Loren Ban, Grite L. Abma, William G. Fortune, Helen H. Fielding, Bruce L. Yoder, Ruth Signorell

**Affiliations:** † Department of Chemistry and Applied Biosciences, ETH Zurich, 8093 Zurich, Switzerland; ‡ Department of Chemistry, University College London, WC1H 0AJ London, U.K.

## Abstract

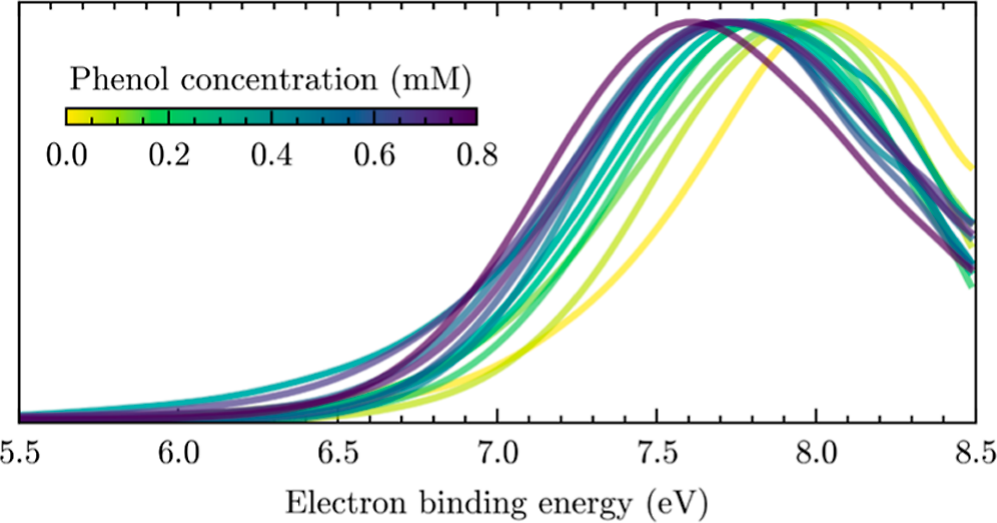

Biochemistry and a large part of atmospheric chemistry occur in aqueous environments or at aqueous interfaces, where (photo)chemical reaction rates can be increased by up to several orders of magnitude. The key to understanding the chemistry and photoresponse of molecules in and “on” water lies in their valence electronic structure, with a sensitive probe being photoelectron spectroscopy. This work reports velocity-map photoelectron imaging of submicrometer-sized aqueous phenol droplets in the valence region after nonresonant (288 nm) and resonance-enhanced (274 nm) two-photon ionization with femtosecond ultraviolet light, complementing previous liquid microjet studies. For nonresonant photoionization, our concentration-dependent study reveals a systematic decrease in the vertical binding energy (VBE) of aqueous phenol from 8.0 ± 0.1 eV at low concentration (0.01 M) to 7.6 ± 0.1 eV at high concentration (0.8 M). We attribute this shift to a systematic lowering of the energy of the lowest cationic state with increasing concentration caused by the phenol dimer and aggregate formation at the droplet surface. Contrary to nonresonant photoionization, no significant concentration dependence of the VBE was observed for resonance-enhanced photoionization. We explain the concentration-independent VBE of ∼8.1 eV observed upon resonant ionization by ultrafast intermediate state relaxation and changes in the accessible Franck–Condon region as a consequence of the lowering of the intermediate state potential energy due to the formation of phenol excimers and excited phenol aggregates. Correcting for the influence of electron transport scattering in the droplets reduced the measured VBEs by 0.1–0.2 eV.

## 1 Introduction

### 1.1 General Background

A large part of nature’s chemistry, particularly atmospheric and biochemistry, occurs in aqueous environments or at aqueous interfaces. Water is also a desirable solvent in synthetic chemistry due to its sustainability, safety, and low cost.^1^ Intriguingly, research in recent years showed how (photo)chemical reactions can be accelerated in small particles and droplets, especially at the air–water interface.^1−12^ This acceleration, which can potentially reach several orders of magnitude, is attributed, for example, to the physical confinement of reagents (amphiphilic surfactants), the partial hydration structure at the interface, electric field effects due to surface charge, and changed pH.^4,6,12^

The key to understanding the chemistry and photoresponse of molecules surrounded by bulk water or at interfaces are the valence electronic structure and photoinduced dynamics for which photoelectron spectroscopy (PES) offers a direct, sensitive probe.^13−16^ The established methods for measuring photoelectron (PE) spectra of liquids are liquid microjets (LJs) coupled to magnetic-bottle or time-of-flight spectrometers.^13−15,17−21^ However, LJ PE spectrometers come with technical challenges such as electrokinetic charging, vacuum level offsets, and uncertainties in instrument function at low electron kinetic energies (eKEs).^22−25^ In addition, the measurement of photoelectron angular distributions (PAD) using LJ PE spectrometers is very time-consuming, making angle-resolved studies difficult. These challenges specific to LJ spectrometers can be circumvented by PE velocity-map imaging (VMI) of aqueous droplets (referred to as droplet-VMI in the following).^15,26,27^ VMI provides the full PAD in a single measurement (angular multiplexing), instead of sequentially recording narrow angular ranges.^15,26,27^ The PAD can provide information on the orbital character of confined systems, droplet size, electron scattering in droplets and liquids,^18,26,28−31^ and as we show below also on the aggregate state of confined systems (aqueous droplets, liquid/solid residual particles after solvent evaporation, and solvent gas phase contributions).

The present work revolves around phenol as a model for amphiphilic organic solutes and common chromophore moiety in biologically relevant photoactive proteins such as the green fluorescent protein, photoactive yellow protein, and photosystem II.^32−34^ We present photoelectron images and spectra of submicron-sized aqueous phenol droplets obtained by ultraviolet (UV) two-photon ionization (2PI), and we show how this droplet approach provides new information about this system that complements the results previously obtained from PES in LJs.^16,21,35−38^

### 1.2 Electronic Structure and Dynamics of Phenol: Gas, Liquid, Droplets, and Interface

**Figure 1:**
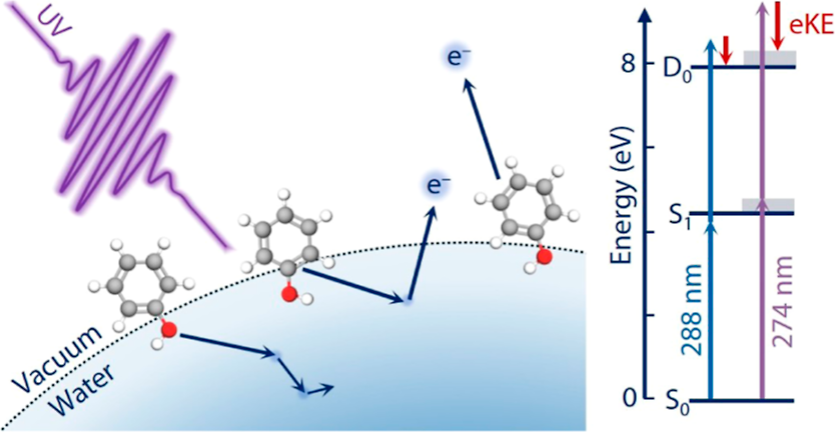
Schematic illustration of the photoionization of phenol molecules at the vacuum–water interface, photoelectron transport scattering and escape (left), and the energy-level diagram of aqueous phenol (right). The vertical arrows represent the employed nonresonant (blue) and 1 + 1 resonance-enhanced (purple) two-photon ionization schemes.

Several studies have addressed the energetics and dynamics of phenol in the gas phase, in solution, and at the air–water interface. The gas-phase ultraviolet–visible (UV–vis) absorption spectrum of phenol is characterized by two bands centered around 270 nm (4.59 eV) and 205 nm (6.05 eV), corresponding to transitions from the S_0_ ground state to the S_1_/1^1^ππ* and S_3_/2^1^ππ* excited states, respectively (Figures 1, S1, and S2).^37^ The optically dark S_2_/1^1^πσ* state lies between the two, is dissociative along the O–H stretch coordinate, and forms conical intersections (CIs) with the S_1_ and S_0_ states. The absorption spectra of phenol in the gas phase and aqueous solution are similar; i.e., the overall excitation energies are barely perturbed. However, the S_1_ band is broadened and loses its structure.^37,39^

Phenol is known to accumulate at the air–water interface due to its amphiphilic character,^40−44^ with only the hydrophilic hydroxyl group immersed and solvated in water and the hydrophobic phenyl ring protruding into the air perpendicular to the interface.^7,10,21,45,46^ The unique solvation environment at the air–water interface can affect the rate of (photo)chemical reactions.^2,7,10^ In bulk aqueous solution, 267 nm excitation to the S_1_/1^1^ππ* state leads to slow formation of PhO^•^, H^+^, and e^–^ on a nanosecond timescale. However, at the air–water interface, this 267 nm photodissociation becomes ultrafast, 10^4^ times faster than that in bulk (<0.1 ps).^7^ Mechanistically, it was proposed that photoexcitation to vibrational levels of the S_1_/1^1^ππ* state above the S_1_/S_2_ CI enables ultrafast photodissociation via internal conversion (IC) to the dissociative S_2_/1^1^πσ* state.^7^ Contrarily, photodissociation following excitation to vibrational levels below the CI is restricted to slow (ns) autoionization from the S_1_ minimum to the PhOH^+^ + e^–^ asymptote, followed by rapid deprotonation to form PhO^•^ + H^+^.^47^ The proposed reason why the same 267 nm photoexcitation causes nanosecond and subpicosecond photodissociation in bulk and at the interface, respectively, is a stabilization of the S_2_/1^1^πσ* state by phenol’s unique partial hydration structure at the interface. The S_2_/1^1^πσ* stabilization leads to a substantial (0.4 eV) lowering of the energy level of the CI, enabling the ultrafast “above CI” photodissociation.^7,46^

Table 1 provides an overview of previously determined values for the first and second vertical binding energy (VBE_1_ and VBE_2_, respectively), corresponding to the transitions from S_0_ to D_0_ (electron hole in HOMO; Figure 1) and from S_0_ to D_1_ (electron hole in HOMO–1), respectively. The VBE is also sometimes referred to as the vertical ionization energy (VIE). For the aqueous phase, the VBE is usually assumed to correspond to the electron binding energy (eBE), where the experimental eBE spectrum has maximal intensity. For gas-phase phenol, VBE_1_ and VBE_2_ were determined to lie in the range of 8.6–8.8 and 9.3–9.7 eV, respectively.^48−57^ As expected, the VBEs of aqueous phenol recorded in LJs are lower by several tenths of an eV than those of gas-phase phenol because the generated radical cationic doublet states are stabilized upon hydration. The most recent value for VBE_1_ of 7.88 eV obtained from nonresonant two-photon ionization (N2PI) using UV light of 290 nm (4.28 eV)^21^ differs from previously reported values but agrees with the value obtained from the nonresonant, single-photon X-ray (*h*ν = 200 eV) PES.^35^ The VBE_1_ from 1 + 1 resonance-enhanced two-photon ionization (R2PI) recorded at resonant UV wavelengths of 275, 278.6, and 272.5 nm lie 0.1, 0.3, and 0.5 eV higher than the value from N2PI, respectively.^21^

**Table 1:** Measured Vertical Binding Energies of Aqueous Phenol Corresponding to Ionization from S_0_ to D_0_ (Electron Hole in HOMO) and D_1_ (Electron Hole in HOMO–1)^a^

method	λ/nm	*c*/M	VBE_1_/eV	VBE_2_/eV
**Gas Phase**				
PES and other^48−57^	div	n/a	8.6–8.8	9.3–9.5
1 + 1 UV PES^37^	235.5	n/a	8.8 ± 0.1	9.7 ± 0.1
**Liquid Jets**				
X-ray PES^35^	6.8	0.8	7.8 ± 0.1	8.6 ± 0.1
UV PES^37^	235.5	0.1	7.6 ± 0.1	8.5 ± 0.1
UV PES^38^	235.5	0.1	8.0 ± 0.1	8.9 ± 0.1
UV PES^21^	290	0.0001	7.88 ± 0.09 (7.76, 7.90^b^)	n/a
1 + 1 UV PES^38^	275	0.1	8.0 ± 0.1	8.4
1 + 1 UV PES^21^	278.6	0.0001	8.20 ± 0.09 (8.17)	n/a
1 + 1 UV PES^21^	272.5	0.0001	8.37 ± 0.09 (8.33, 8.37^b^)	n/a
**Droplets**				
VUV PES^45^	50	0.2–0.5	8.67 ± 0.05	9.46 ± 0.05
UV PES (this work)	288	0.01	8.0 ± 0.1 (7.9)	n/a
UV PES (this work)	288	0.8	7.6 ± 0.1 (7.4)	n/a
1 + 1 UV PES (this work)	274	0.01–0.8	∼8.1 ± 0.1 (∼8.0)	n/a

a The values in parentheses correspond to genuine VBEs, i.e., VBEs corrected for electron scattering.

b These values are obtained from a new retrieval (publication in preparation).

Remarkably, a previous droplet PES study using vacuum ultraviolet (VUV) light of *h*ν = 25 eV reported a VBE_1_ of 8.67 eV and a VBE_2_ of 9.46 eV for aqueous phase phenol,^45^ values equal to the gas-phase values and around 1 eV higher than the LJ values. Both the agreement of VBEs in the aqueous droplet phase with the corresponding gas phase values and the large difference between the droplet and LJ values are surprising, even considering that the droplet and LJ results may differ slightly. One of the challenging parts in droplet PES is the transfer of aqueous droplets with high vapor pressure from the aerosol source into a vacuum. Significant water evaporation must be avoided; otherwise, only gaseous species and/or (in the case of low- and medium-volatility solutes) residual dry solute droplets/particles reach the ionization region instead of liquid droplets.

In this work, we report the first droplet-VMI measurements of aqueous phenol. We used our droplet VMI photoelectron spectrometer for these studies, the design of which has been optimized to avoid significant water evaporation.^15,26,27,58,59^ The VMI capability of our spectrometer was essential to provide clear proof that we probed aqueous phenol droplets and not residual gas phase or dried liquid phenol droplets. We focused on the phenol concentration dependence of the VBE_1_ obtained upon N2PI with UV light at 288 nm (4.31 eV) and upon R2PI with UV light at 274 nm (4.53 eV) and on the comparison between nonresonant (N2PI) and resonant (R2PI) two-photon UV ionization. Using our detailed electron scattering model,^15,18,26,31^ we also address the question of how strongly the measured VBE is influenced by electron scattering in the droplets.

## 2 Experimental and Computational Methods

### 2.1 Droplet VMI Photoelectron Spectrometer

Data were collected using a droplet VMI photoelectron spectrometer coupled to an aerosol droplet generation and conditioning unit and a femtosecond laser system. A sketch of the setup is provided in Figure S3, and details of the setup are provided in refs (26, 28, 58, 60, and 61).

Aqueous submicrometer-sized droplets were generated *ex vacuo* by atomizing aqueous phenol solution (0.01–0.8 M) using a commercial Collison-type atomizer (3076, TSI Inc.). Before entering the photoelectron spectrometer, the aqueous phenol droplets may be charge neutralized (net neutral electric charge) using a soft X-ray bipolar diffusion charger (3088, TSI Inc.) or dried with a silica-based diffusion dryer. Charge neutralization allowed us to study the potential influence of electric charges on the VBEs, and drying was used to produce droplets of near-neat liquid phenol (Section 2.3). The droplets were collimated into a droplet beam and transferred into vacuum (∼10^–4^ mbar, first vacuum chamber of the photoelectron spectrometer in Figure S3) with a home-built aerodynamic lens stack (ADL). Differential pumping was used to maintain pressures ≲10^–6^ mbar in the detection chamber when the aerosol beam was on. Transporting aqueous droplets with high vapor pressure is experimentally challenging because of potential evaporation, which occurs mainly in the region before transfer into a vacuum. Once in vacuum, the droplets rapidly become supercooled due to evaporative cooling, preventing major further evaporation.^45,62−64^ For an average droplet radius of about 250 nm (Section 2.3), supercooling in a vacuum down to 240 K takes place within a few tens of microseconds and results in a radius change of the droplet of less than 4%. Our experimental setup (ADL design, tubings, travel time, and relative humidity) has been optimized to avoid significant droplet evaporation.

In the detection chamber, the droplets were ionized (2PI) at photon energies of 4.31 eV (288 nm, N2PI) or 4.53 eV (274 nm, R2PI) from a frequency-tunable, pulsed femtosecond UV laser (1 kHz repetition rate, ≲80 fs pulse duration). The UV pulses were generated by an optical parametric amplifier (Opera, Coherent) seeded with an 800 nm femtosecond (≲40 fs) Ti:Sa oscillator operating at 1 kHz (Astrella, Coherent). The polarization direction was perpendicular to the time-of-flight axis. The emitted PEs were analyzed by VMI, i.e., the generated three-dimensional photoelectron velocity distribution was projected onto a two-dimensional imaging detector using an electrostatic lens. The imaging detector consisted of a gated position-sensitive microchannel plate (fast high-voltage switch, 200 ns gate), a fast phosphor screen, and a kHz camera that recorded the two-dimensional projections on a single-laser-shot basis (500 Hz). The VMI spectrometer was calibrated with the 2 + 1 resonance-enhanced multiphoton ionization (REMPI) spectrum of Xe at 250 nm. The PE images contain information about the PAD and the eKE. The eKE spectra were retrieved from the time-averaged VMIs after reconstruction along the laser propagation direction with standard methods.^65^ Note that in droplet VMI-PES, reconstruction is performed along the laser propagation instead of the polarization direction as this is the only cylindrical symmetry axis of the PE cloud.^27^ The eBE spectra were obtained from the eKE spectra using
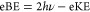
1

The VBEs were assumed to correspond to the eBE values at the maxima of the eBE spectra.

### 2.2 Electron Transport Scattering Simulations and Light Focusing

Inelastic electron scattering of the photoelectrons in liquid before the escape into vacuum results in differences between the measured PADs and eKE spectra and the “genuine” PADs and “genuine” eKE spectra (see refs (15, 18, and 26) for details). The term “genuine” refers to the PADs and eKE spectra one would record in the solvent at the location where photoexcitation took place, i.e., before transport electron scattering could have occurred. Recording such PADs and spectra is not possible. In contrast to the genuine properties, measured PADs and eKE spectra are always modified by electron scattering in the liquid and hence differ from their genuine values. The difference between the measured and genuine properties depends on the eKE because electron scattering depends on the eKE. Generally, scattering results in more isotropic PADs and shifted (to lower eKEs) and distorted (asymmetric band broadening) eKE spectra. To retrieve unperturbed, genuine PADs and eKE spectra, measured PADs and eKE spectra must be corrected for electron scattering. This results in genuine VBEs, which can be compared even when recorded under very different conditions (e.g., different eKEs). In recent years, approaches with different levels of detail have been presented to obtain genuine PE spectra.^18,21,26,66^ Another important aspect that has often been ignored is the focusing of light in droplets and LJs (Figure S7 top row). Such resonance phenomena can result in a strongly inhomogeneous distribution of the light intensity within droplets and LJs, which in turn affects measured PADs and eKE spectra.^15,18,26^

Here, we used a detailed model to simulate photoelectron images (i.e., PADs and eKE spectra) and to retrieve genuine eKE spectra and VBEs. The model takes into account inhomogeneities of light intensity within the droplets (here mainly nanofocusing, Figure S7 top row), treats the influence of electron scattering in detail, and accurately reproduces the experimental setup, settings, and detection (VMI). For details, we refer the reader to our previous publications and the corresponding Supporting Information.^15,18,26,31^ Briefly, the droplet’s internal light intensity was obtained from a numerical solution of Maxwell’s equations using the Amsterdam Discrete Dipole Approximation (ADDA) code.^67^ We assumed spherical droplets irradiated by plane-wave light. The employed wavelength- and concentration-dependent complex refractive indices, *N* = *n* + *ik*, are given in Table S1. The probability of generating a photoelectron by two-photon ionization at a certain location in the droplet is proportional to the square of the light intensity at this location. In accordance with Riley et al.,^37^ we used a genuine PAD with a single anisotropy parameter of β_2_ = 1. The genuine eKE band was modeled by a Gaussian function and obtained from a fit of the calculated VMI to the measured VMIs (analogous to ref (18)). The probabilistic electron scattering model (Monte–Carlo solution of the transport equation) was formulated as a random walk with an exponential distribution of step lengths with a mean step length corresponding to the inverse of the total scattering cross section weighted by the number density of water molecules. The distributions of energy loss and scattering angle were given by the differential scattering cross sections of the individual scattering channels for water (elastic and all inelastic phonon, vibron, and electronic channels) from ref (31). An escape barrier at the droplet surface of |*V*_0_| = 1.0 eV was assumed, with conservation of angular momentum for the escape at the surface (Snell’s law with inelastic forward scattering). The accumulation of phenol at the droplet surface was modeled by a Gaussian concentration distribution (Figure S4) in accordance with recent neutron reflectivity experiments and molecular dynamics simulations.^21,40,41,44^ The VMI conditions for the projection of the electrons onto the electron detector mimic the experimental conditions. Image reconstruction to retrieve the eKE spectrum was identical with the one used for experimental images (see above).

### 2.3 Detection of Aqueous Phenol Droplets and In Situ Determination of Droplet Size

Below, we provide clear experimental evidence that we studied aqueous phenol droplets and not residual dried, pure phenol droplets, or phenol gas phase, and we estimate the droplet size in situ where photoionization occurs. The PAD is crucial in this context as it also offers an alternative approach to in situ droplet size determination. As mentioned above, transporting aqueous droplets while avoiding evaporation is challenging and requires careful design of aerosol transport on the “air side” and of the ADL and control of the conditions. Evaporation is also the primary reason why commercial aerosol instrumentation for particle sizing, such as scanning particle mobility sizers, is not suitable for water droplet sizing.^68^ Furthermore, in situ sizing is important not only due to evaporation but also because of possible droplet coagulation during transport.

#### 2.3.1 Proof of Aqueous Droplets

**Figure 2:**
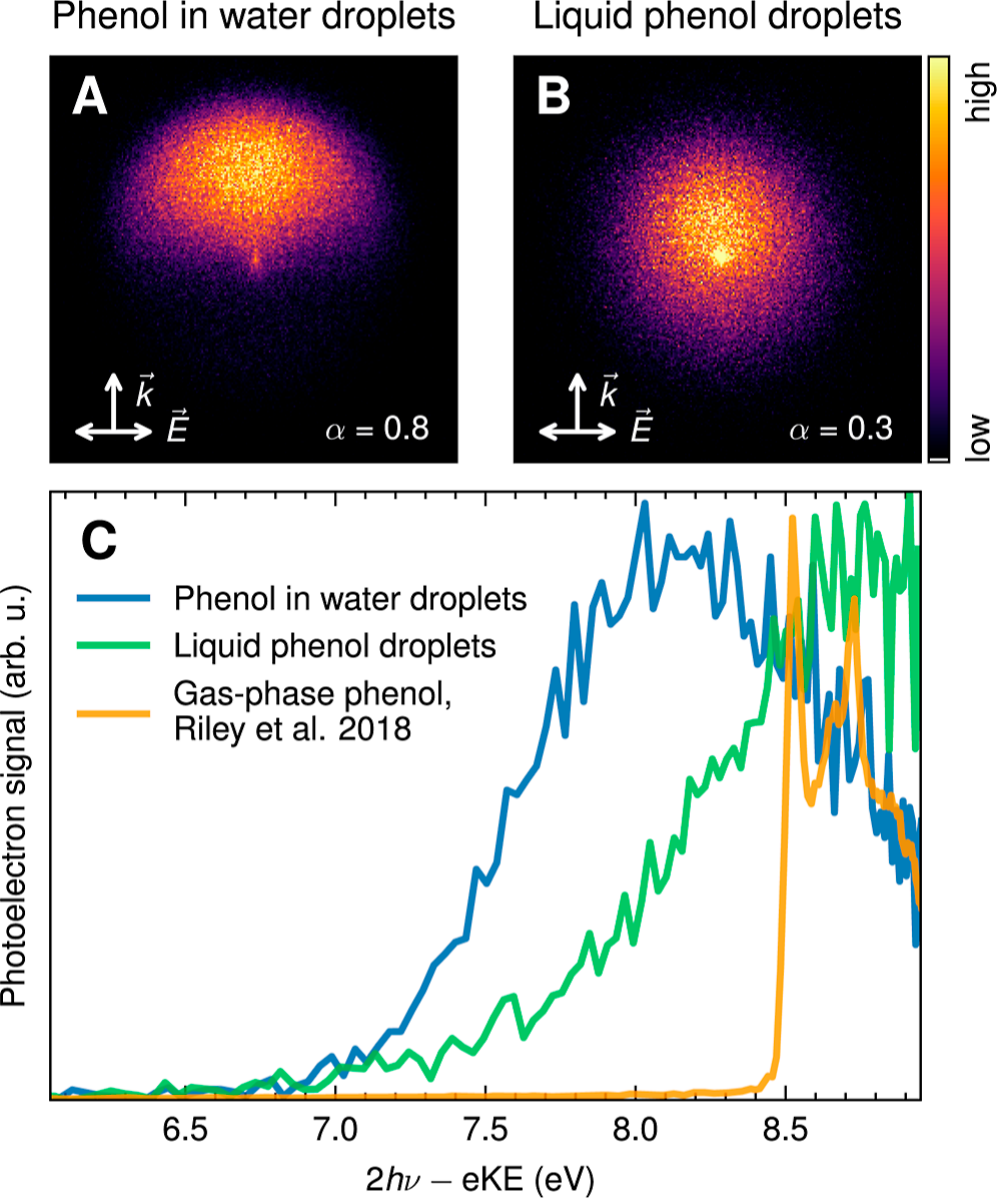
Photoelectron VMIs (A,B) and corresponding photoelectron spectra (C) recorded after two-photon ionization at 274 nm for aqueous phenol droplets [(A), blue spectrum in (C)] and neat liquid phenol droplets [(B), green spectrum in (C)] generated by drying the aqueous droplets. The 1 + 1 REMPI photoelectron spectrum of gas-phase phenol at 275 nm from Riley et al.^37^ (orange) is shown as a reference. (Adapted with permission from ref (37). Copyright 2018 American Chemical Society.) The arrows indicate laser propagation  and polarization  direction.

The top row in Figure 2 shows VMIs of aqueous phenol droplets (A) and of near-pure phenol droplets with pronounced contributions from the phenol gas phase (B). Near-pure phenol droplets were generated by drying aqueous phenol droplets with a diffusion drier placed directly after the droplet generation device. Since it is likely that drying will not remove all water from the droplets, we assume that the pure phenol droplets are still liquid and not solid. The aqueous phenol droplet VMI shows a pronounced asymmetry along the laser propagation direction, meaning that most of the electrons in the droplet are generated on the side opposite the incident light, while only a few electrons are generated on the side facing the incident light. This asymmetry in the VMI arises because the droplets act as optical resonators (nanofocusing) and reflect the asymmetry of the internal light intensity distribution because of nanofocusing due to the high water content in the droplets (Figures S4 and S7).^26,27^ Important here is that such a pronounced asymmetry occurs only for aqueous phenol droplets but not for pure phenol droplets or for phenol vapor. The VMI of pure phenol droplets shows a much less pronounced forward–backward asymmetry (Figure 2B). Nanofocusing is strongly dampened in neat phenol droplets due to the pronounced light absorption of pure phenol compared with aqueous solutions or neat water and the substantially smaller size (70 nm radius) of the pure phenol droplets compared with that of the aqueous phenol droplets (250 nm). Nanofocusing is a light resonance phenomenon that can occur only in droplets/particles but not in gas phase molecules. VMIs of the latter do not show any forward–backward asymmetry. The pronounced forward–backward asymmetry of the aqueous droplets is thus clear evidence of their aqueous character. Furthermore, we can also detect pure water droplets (no phenol), which have an even higher vapor pressure than aqueous phenol droplets (see PES of neat water in Figure S5), demonstrating that even relatively volatile droplets reach the ionization region. Since two-photon ionization does not suffice to ionize pure water droplets (VBE_1b1_ ∼ 11.3 eV)^20,21^, we increased the laser power to enhance three-photon ionization of neat water for the spectra in Figure S5.

Time-of-flight ion mass spectrometry offers yet another way to examine the presence of water in the droplets. We irradiated the droplets with focused femtosecond laser pulses at a wavelength of 800 nm. The high light intensity due to focusing of the laser, together with nanofocusing within the droplets, leads to a partial disintegration of the droplets and thus to emission of water cluster ions.^69^ The observation of such water cluster ions in the mass spectrum in Figure S6 provides additional evidence for the presence of aqueous droplets and speaks clearly against dried, liquid phenol droplets.

#### 2.3.2 In Situ Droplet Sizing

We found that the forward–backward asymmetry is essentially independent of the chosen concentration (0.01–0.8 M) of the aqueous phenol droplets and the chosen laser wavelength (288 or 274 nm) since the corresponding refractive index changes lead to only negligible changes in the degree of nanofocusing. In other words, this means that the forward–backward asymmetry is determined almost exclusively by the droplet size, thus providing a possibility to determine the droplet size directly from the asymmetry of the VMIs. To this end, we quantified the forward–backward asymmetry in the VMIs along the laser propagation direction  by the parameter^70^
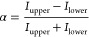
2where *I*_upper_ and *I*_lower_ are the total electron intensities of the upper and lower halves of the images (Figure 2).

We found an α value of about 0.8 for all aqueous phenol droplets. To determine the droplet size, we then simulated VMIs for different droplet radii between 25 and 500 nm, calculated α for all of these images, and compared the calculated α values with the experimental value of α ∼ 0.8. Figure S7 shows that the comparison results in an average droplet radius of about 250 nm. This value was later used for all of the further VMI simulations.

## 3 Results and Discussion

### 3.1 Measured PE Spectra and Retrieval of Genuine VBE_1_

**Figure 3:**
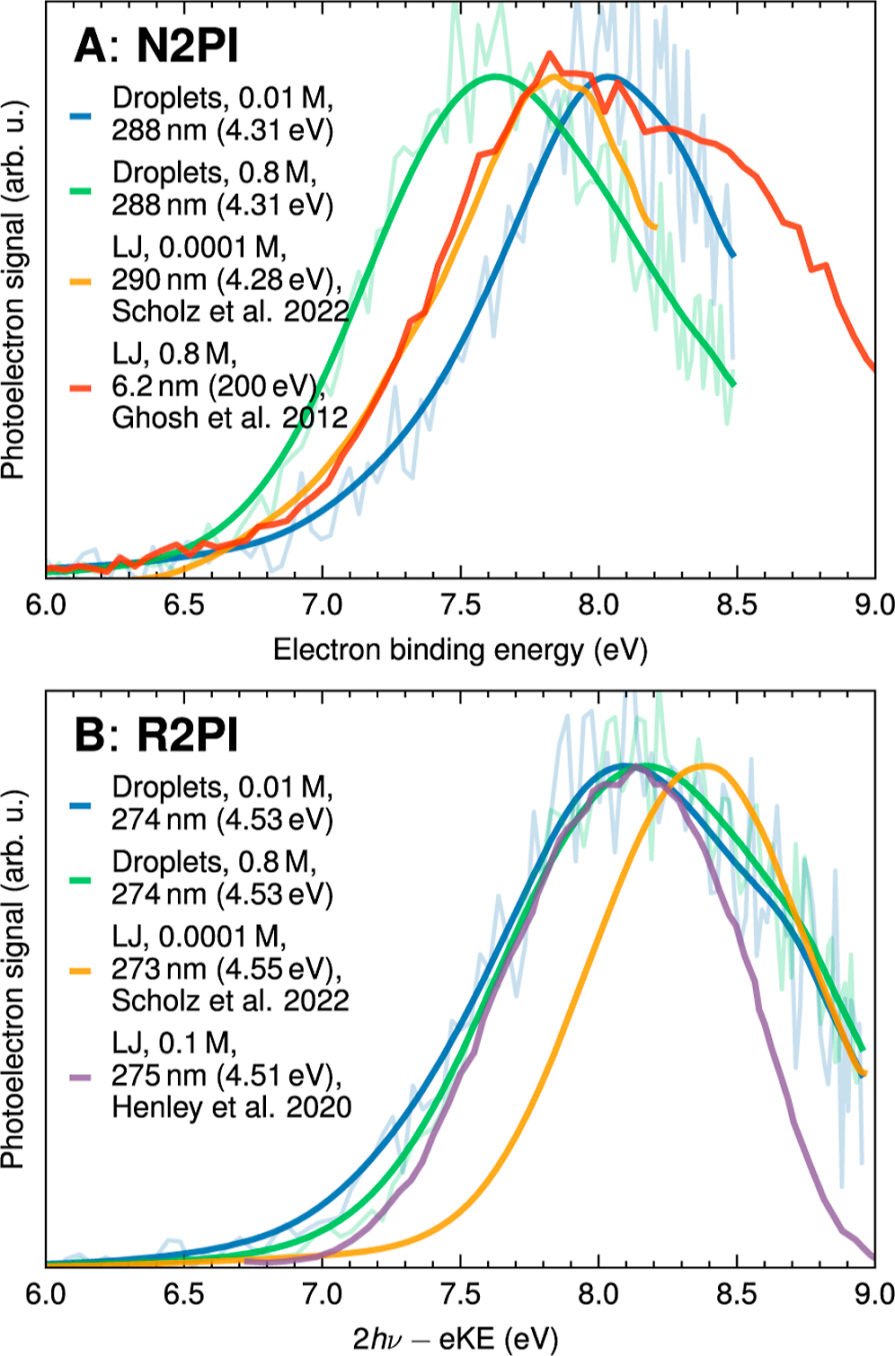
Experimental photoelectron spectra of aqueous phenol in submicrometer-sized water droplets recorded after nonresonant two-photon ionization (N2PI) at 288 nm (top panel) and 1 + 1 resonance-enhanced two-photon ionization (R2PI) at 274 nm (bottom panel). The concentrations of the atomized bulk aqueous phenol solutions are 0.01 M (blue) and 0.8 M (green). Literature photoelectron spectra of phenol in liquid-water microjets are shown for comparison.^21,35,38^ To enhance clarity, a binomial smoothing routine was applied to the droplet spectra and the LJ spectra from Scholz et al.^21^ The LJ spectra from refs (35 and 38) are digitized versions from the publications. Adapted with permission from ref (38). Copyright 2020 Royal Society, licensed under a Creative Commons Attribution 3.0 Unported License. Adapted with permission from refs (21 and 35). Copyright 2012 and 2022 American Chemical Society.

**Figure 4:**
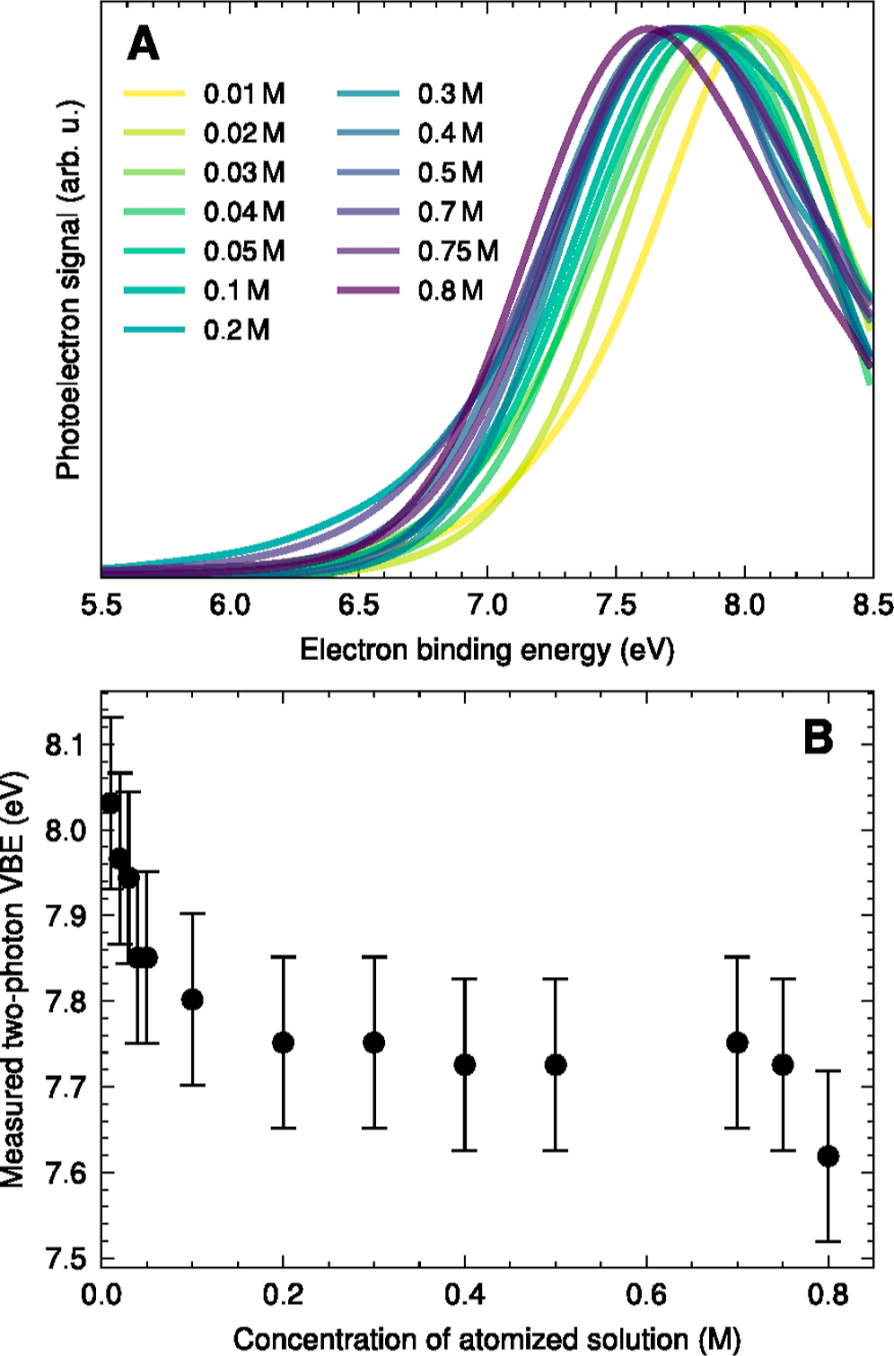
(A) Experimental N2PI droplet photoelectron spectra as a function of the phenol concentration (legend). For clarity, a binomial smoothing routine was applied to the raw spectra. (B) Concentration dependence of VBE_1_. The error bars indicate the estimated overall uncertainty of the determination of VBE_1_.

Figure 3A compares experimental N2PI eBE spectra recorded at 288 nm for the lowest (0.01 M, blue line) and highest (0.8 M, green line) phenol concentration. The N2PI spectra predominantly map out the S_0_ → D_0_ transition (Figure 1). For all spectra, the very weak water background resulting from the three-photon ionization of water was taken into account by subtracting a water spectrum (see caption of Figure S5). The VBE_1_ decreases by ∼0.4 eV from 8.0 ± 0.1 eV at the lowest concentration to 7.6 ± 0.1 eV at the highest concentration. This decrease in VBE_1_ corresponds to an increase in the eKE (eq 1). Figure 4A reveals a systematic trend toward lower VBE_1_ with increasing phenol concentrations. The decrease is pronounced up to a concentration of about 0.2 M and then levels off at even higher concentrations.

For resonance-enhanced photoionization (R2PI at 274 nm; Figure 3B), by contrast, practically no concentration dependency of the PE spectrum was observed within our experimental uncertainty. Across all concentrations, VBE_1_ has a value of approximately 8.1 eV. This virtually coincides with the VBE of 8.0 eV for N2PI at the lowest phenol concentrations but is ∼0.5 eV higher than the N2PI VBE_1_ of 7.6 eV found at the highest phenol concentration. In the case of R2PI, the first photon prepares aqueous phenol in its S_1_/1^1^ππ* state. The subsequent photon then ionized the photoexcited phenol. Therefore, the R2PI spectra predominantly map out the S_1_ → D_0_ transition (Figure 1).

**Figure 5:**
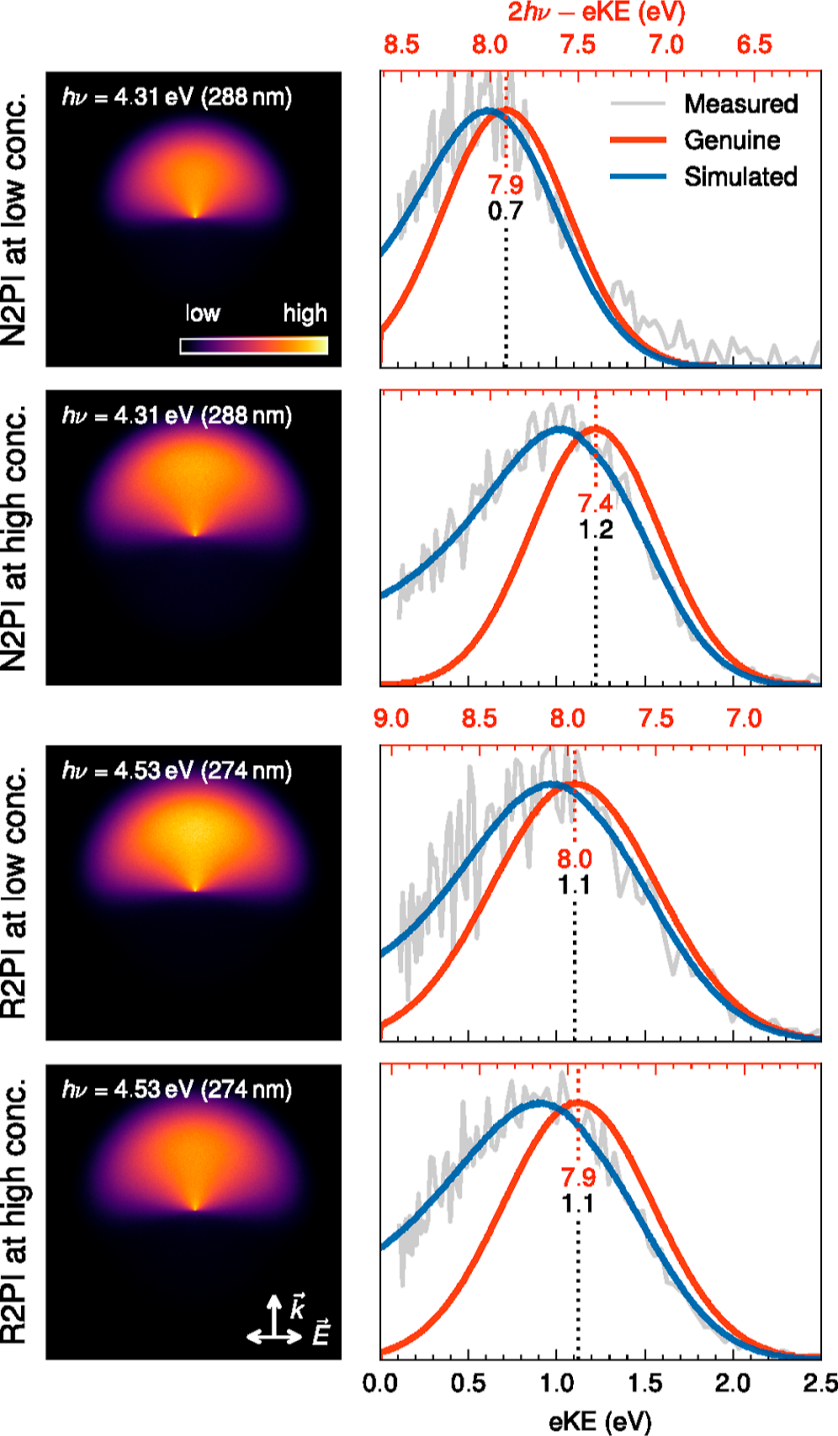
Left: Simulated photoelectron VMIs. Arrows indicate laser propagation  and polarization direction . Right: Experimental (gray), simulated (blue), and genuine (red) photoelectron spectra. Top row: N2PI of droplets at 0.01 M phenol concentration. Second row: N2PI of droplets at 0.8 M phenol concentration. Third row: R2PI of droplets at 0.01 M phenol concentration. Bottom row: R2PI of droplets at 0.8 M phenol concentration. The lower abscissa in black shows the eKEs and the upper abscissa in red shows the eBEs. The vertical dotted lines indicate the genuine VBE_1_ (red number) and the corresponding genuine eKE (black number) in eV. See text and Figure S4 for more information.

To determine genuine VBE_1_, i.e., values that are not modified by electron transport scattering in the droplet, we performed fits to the measured VMIs, as explained in Section 2.3. This also provides genuine eBE (eKE) spectra, i.e., eBE (eKE) spectra free of distortions from scattering. The results are summarized in Figure 5 (see also S4), and the measured and genuine values for VBE_1_ for N2PI at the highest and lowest concentration and for R2PI (concentration independent) are listed in Table 1. The simulated VMIs (Figure 5 left) have the same asymmetry as the experimental VMIs (Figure 2A), and the simulated eBE (eKE) spectra (blue lines in Figure 5 right) match the corresponding measured spectra (gray lines). The red lines show genuine eBE (eKE) spectra. Comparison of the red and blue lines reveals that the VBE_1_ are only slightly influenced by electron scattering. Compared with the measured values, they shift by about 0.1–0.2 eV to lower values, i.e., for N2PI to 7.9 eV at the lowest concentration and 7.4 eV at the highest concentration and for R2PI to ∼8.0 eV (Table 1). The simulations also show that single Gaussian-shaped genuine eBE spectra (red lines) and electron scattering reproduce the nonsymmetric shape of the measured eBE spectra well (agreement between gray and blue lines). This illustrates that in the current case, the nonsymmetric band shape mainly results from electron scattering and not from multiple transitions that would require a multiple Gaussian representation of the genuine spectrum. In general, this highlights that a proper treatment of electron scattering can be important to answer the question of single or multiple transitions.

### 3.2 Comparison with Previous PE Spectra

The range of values of our droplet N2PI VBE_1_ (7.6–8.0 eV) agrees well with those obtained from LJ N2PI (between 7.6 and 8.0 eV)^21,37,38^ and LJ X-ray photoionization (7.8 eV)^35,36^. All of these values, however, are substantially lower than the previously reported droplet VUV value (8.67 eV)^45^. Strikingly, the latter agrees with the gas phase value (Table 1). This raises the question of whether the species probed by VUV PES were actually aqueous droplets and not gas-phase phenol combined with residual dried phenol droplets. Figure 2 shows that the eBE spectrum of pure liquid phenol droplets (green line, generated by drying aqueous phenol droplets) is actually composed of two contributions: a contribution from dried, pure phenol droplets (signal between ∼7 and 8.3 eV) and a substantial contribution from gas-phase phenol (signal above ∼8.3 eV), which agrees with the gas-phase phenol spectrum (orange line) from Riley et al.^37^ The phenol droplet spectrum (green line) also shows that the VBE_1_ of such a composite droplet/gas-phase spectrum lies around 8.7 eV; i.e., it is dominated by the gas-phase contribution. A possible explanation for the significant deviation of the VBE_1_ value of the droplet VUV PES study^45^ from all other studies could be that this study actually examined a sample consisting of particles and gas phase and not of aqueous phenol droplets. Note that in ref (45), no PAD was recorded and no in situ sizing was performed that could provide evidence for aqueous phenol droplets.

The range of values of our droplet N2PI VBE_1_ agrees well with previous LJ studies (Table 1). However, none of the previous studies recorded concentration-dependent VBE_1_. In this context, we note that droplet spectra and LJ spectra recorded at the same concentration of the bulk solution cannot be directly compared because the process of phenol accumulation at the surface is expected to differ in the two samples.^40−44^ The surface-to-volume ratio of a droplet with a radius of 250 nm is about 60 times higher than the surface-to-volume ratio of an LJ with a radius of 10 μm. Assuming that all phenol molecules accumulate in the surface area, the same surface coverage as in the droplets would already be achieved in the LJ at a concentration 60-fold lower than that of the droplets. However, this hypothetical scenario does not account for the very different diffusion timescales to reach complete surface coverage of phenol in the droplets (∼10 μs) compared to LJs (∼100 ms). While in droplets, the time between the formation of the droplets and ionization is sufficient for all phenol molecules to diffuse to the surface, this is not the case in LJs. Furthermore, accumulation of all phenol molecules in the surface layer is physically not very plausible, and the concentration profile within LJs and droplets might differ and depend on the exact conditions. Other parameters that could also influence the value of the VBEs in LJs are, for instance, different light polarizations, different probing angles, electrokinetic charging of the LJ, and addition of alkali halides to LJs. We have investigated the influence of electric charging and addition of alkali halides on droplet spectra (Figures S8 and S9). Since essentially no effect was observed, we assume that neither charging of LJs nor adding alkali halides to LJs should result in differences in comparison with droplet spectra.

For both droplets and LJs, the R2PI VBE_1_ values are generally a few tenths of an eV higher (between 8.1 and 8.4 eV) than the N2PI values, with the exception of the lowest concentration in the droplets. We found that the R2PI VBE_1_ values in the droplet spectra are concentration independent (Table 1, Figure 3B). As already mentioned, the N2PI spectra predominantly map out the S_0_ → D_0_ transition while the R2PI spectra predominantly map out the S_1_ → D_0_ transition (Figure 1). In the case of R2PI, the first photon prepares aqueous phenol in its S_1_/1^1^ππ* state. The subsequent photon then ionizes the photoexcited phenol. The higher value of the R2PI VBE_1_ compared with the N2PI value corresponds to a lower eKE recorded for R2PI compared to N2PI. This difference could be explained by fast intermediate state relaxation in the case of R2PI. As pointed out in the next section, there is no significant effect of ultrafast relaxation on the R2PI droplet spectra at the very lowest concentration. The effect of ultrafast relaxation in the resonant intermediate state gradually grows with increasing concentration and compensates for the lowering of D_0_. This suggests that both effects have the same origin, i.e., increased intermolecular interactions by excimer formation. With a duration of our pulses of ∼80 fs (droplets) and ∼150 fs (LJ), fast relaxation pathways are required. IVR within the S_1_ or changing Franck–Condon profiles were recently suggested as relaxation pathway after excitation with femtosecond lasers.^21^ It remains unclear whether or not electronic relaxation pathways after R2PI via the S_1_/S_2_ (1^1^ππ*/1^1^πσ*) CI are accessible with 274 nm (4.53 eV) light (Figure S1),^7,46^ which might result in fast relaxation. Other fast electronic relaxation pathways can also not be ruled out. We finally note that for N2PI at 288 nm (4.31 eV), the S_1_ cannot be reached because the adiabatic excitation energy (AEE) of the S_0_ → S_1_ transition is estimated to be ≳4.45 eV (Figures 1, S1, and S2).^38,71,72^

### 3.3 Concentration Dependence

**Figure 6:**
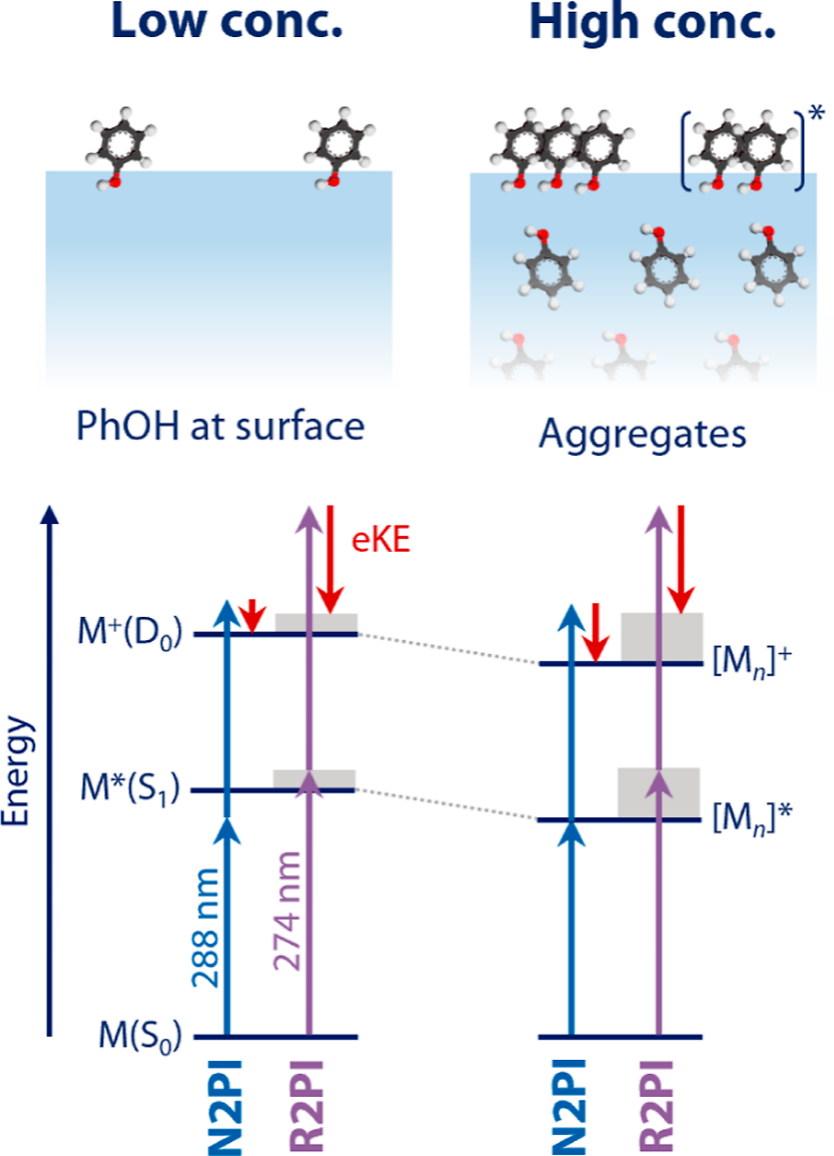
Schematic illustration of the droplet–vacuum interface (top row) and energy-level diagrams and ionization schemes (bottom row) at low (left) and high (right) phenol concentrations. The formation of phenol excimers and excited phenol aggregates at high concentration results in a stabilization of the S_1_ intermediate state and the D_0_ cationic ground state.

In addition to comparing R2PI and N2PI in LJs and droplets, we also investigate the concentration dependence of the droplet VBE_1_, which provides new insight into the ionization of aqueous phenol at the liquid–gas interface and into the dynamics of nonresonant versus resonant excitation. At the lowest concentration (0.01 M), N2PI and R2PI VBE_1_ are equal within uncertainties (∼8.0 ± 0.1 eV, Table 1). If all phenol molecules in the droplet accumulated on the droplet surface, the surface coverage would correspond to only about 7% of a complete monolayer (assuming a molecular volume of phenol of 0.15 nm^3^)^73^. In reality, the surface coverage is likely to be lower, confirming that at a concentration of 0.01 M, the surface molecules can be viewed as individually dissolved phenol molecules that do not interact with other phenol molecules (Figure 6, left). The N2PI VBE_1_ of 8.0 eV thus represents the VBE of the individually dissolved phenol molecules at the droplet surface. Furthermore, the almost identical values of N2PI and R2PI VBE_1_ imply that the intermediate state relaxation in R2PI is essentially negligible at this low concentration for an excitation energy of 4.53 eV (274 nm).

Increasing the concentration of the bulk solution from 0.01 to approximately 0.2 M results in an increasing phenol density at the surface, i.e., a reduction of the phenol–phenol distance and thus an increase in phenol–phenol interactions (Figure 6, right). Our simple surface coverage model predicts the formation of a complete monolayer at a concentration of about 0.14 M, in agreement with previous bulk studies that found phenol monolayers at the vacuum–solution interface at concentrations up to 0.2 M before forming multilayers at higher concentrations.^40,41^ (Here, it should be noted again that the concentration values of our simple model are lower estimates as in reality, not all phenol molecules accumulate at the surface. The partitioning of solute molecules in small droplets might also differ from that in extended systems.) The concentration range from 0.01 to about 0.8 M coincides with the region where the N2PI VBE_1_ decreases by around 0.4 eV from ∼8.0 to ∼7.6 eV (Figure 4B). We propose that this systematic decrease is caused by the formation of phenol dimers and larger aggregates with increasing concentration (Figure 6 right), likely with π-stacked geometries. Aggregate formation was observed for many neat, solvated, and interfacial aromatic systems.^74−79^ It is also known for benzene dimers, for example, that dimer formation stabilizes the cation by several tenths of an eV relative to the neutral dimer, resulting in an ionization energy of the dimer that lies several tenths of an eV below that of the monomer.^75,80−82^ Based on these arguments, we assign the systematic decrease of the N2PI VBE_1_ to a systematic lowering of the cationic D_0_ state due to increasing formation of phenol dimers and aggregates (Figure 6).

The N2PI VBE_1_ decreases steeply up to 0.2 M, whereas the change in VBE_1_ is more subtle at higher concentrations (Figure 4B) where the range of phenol multilayer formation in the surface area is reached.^40,41^ This suggests that the main effect is already captured by the formation of a more or less complete monolayer, consistent with the assumption that the π stacking of phenol molecules is the main factor. At the highest concentration, a maximum of about six multilayers could theoretically be formed (assuming all molecules accumulate close to the surface). The corresponding, theoretical multilayer thickness of ∼6 nm (assuming a monolayer thicknesses of around 1 nm)^21,40,46^ exceeds the probing depth for photoelectrons of ∼4 nm (1/e probing depth for eKEs around 1 eV)^18^, i.e., only the first ∼4 nm layers contribute to the PE signal at concentrations above ∼0.5 M.

Phenol dimer and aggregate formation lower not only the D_0_ ionic state but also the S_1_ state (Figure 6). Substantial shifts of the S_1_ potential energy up to several tenths of an eV were observed in aromatic systems due to excimer and excited aggregate formation.^74,75,77−79^ As mentioned above, the agreement of R2PI with N2PI VBE_1_ at the lowest concentration (0.01 M) implies that there is no substantial intermediate-state relaxation in S_1_ in the case of R2PI in the absence of excimer and excited aggregate formation. Their formation at higher concentrations, by contrast, could substantially enhance intermediate state relaxation in the S_1_, thus explaining the insensitivity of the R2PI VBE_1_ to the phenol concentration (Figure 3B, Table 1). In the case of R2PI, all the energy “gained” by the lowering of the D_0_ state through intermolecular interaction ends up as internal excitation of the ion and not as kinetic energy of the photoelectron, in contrast to N2PI. Part of that energy might also be dissipated to the solvent, but given the femtosecond timescale of the R2PI, this relaxation channel is likely too slow to be significant. Such a contribution would not change the argument because the corresponding energy would still be lost for the photoelectron. There are two ways in which the intermolecular interaction energy ends up in the ion in the case of R2PI. One is fast intermediate-state relaxation (IVR, IC, etc.), and the other is a change of the Franck–Condon region accessed by the second photon as a consequence of a modified intermediate state potential energy surface. In both cases, the origin of the effect is the shift of the S_1_ resulting from excimer and excited aggregate formation. An assessment of the relative contribution of the two pathways would require extensive quantum chemical calculations to explore the potential energy surfaces of all electronic states involved combined with appropriate modeling of Franck–Condon envelopes.

## 4 Conclusions

Photoelectron VMI of submicrometer aqueous droplets benefits from well-defined, confined droplet volume, high surface area to volume ratio, angle multiplexing to record PADs, and from the fact that electrically neutral and charged droplets can be studied. The present study illustrates its advantages for the study of aqueous phenol droplets with submicrometer sizes after nonresonant (N2PI) and resonance-enhanced (R2PI) two-photon ionization using femtosecond UV light of 4.31 and 4.53 eV, respectively. The droplet radius (∼250 nm) was determined in situ from experimental VMIs, and the influence of electron transport scattering within the droplets was considered using a detailed electron scattering model.

The focus was on the concentration dependence of the vertical binding energy (VBE) after N2PI and R2PI. Both excitation schemes produce phenol cations in the D_0_ ground state. Phenol accumulates at the vacuum–water interface, allowing the study of increasing phenol surface coverage with increasing solution concentration. For N2PI, the observed decrease in VBE from 8.0 to 7.6 eV with an increasing concentration reflects the decrease in energy of the D_0_ state. We attribute this decrease to the formation of phenol dimers and larger aggregates with π-stacked geometries on the droplet surface with an increasing concentration. This would also explain the observation of a strong decrease in VBE during the formation of the first complete monolayer and the relatively small change in VBE as further layers (multilayers) form. Other effects, such as the build-up of small electrostatic surface potential by molecular dipole alignment, are likely of minor importance. The formation of phenol aggregates at the droplet surface also alters the phenol–water interaction, which may also impact the VBE. However, in contrast to the formation of phenol aggregates, altered phenol–water interaction is unlikely to play a major role in the concentration-dependent VBE shift. The dominance of phenol–phenol interactions is also reflected by the observed concentration-independence of the VBE from R2PI. A VBE of ∼8.1 eV was measured for all concentrations examined. The formation of phenol aggregates stabilizes not only the D_0_ ionic ground state but also the S_1_ intermediate state, i.e., the state resonantly excited by the first photon. Fast intermediate state relaxation and a change in the Franck–Condon region accessed by the second photon could explain why, in the case of R2PI, all excess energy gained by the decrease of the D_0_ energy due to phenol aggregate formation ends up as the internal energy of the ion and not as eKE. Clarification of this hypothesis for such a large system would require extensive quantum chemical vibronic calculations beyond the scope of the present experimental study.

## Supporting Information Available

Schematic potential energy curves for the photodissociation of aqueous phenol; UV–vis spectrum of aqueous phenol and laser bandwidths; experimental setup; complex refractive indices; extended Figure 5; water backgrounds; TOF spectra; in situ size determination, and effect of electric charges and added alkali halides on the PE spectra (PDF)

## Notes

Published as part of *The Journal of Physical Chemistry A*special issue “Richard J. Saykally Festschrift”.
