# Supplementary material for: Valence Electronic Structure of Interfacial Phenol in Water Droplets
Source: J Phys Chem A. 2024 Aug 25;128(35):7396–406. doi: 10.1021/acs.jpca.4c04269 (PMC11382284; doi:10.1021/acs.jpca.4c04269)
Supplement: Supplementary file 1 — jp4c04269_si_001.pdf [file jp4c04269_si_001.pdf]

Supporting Information

Valence Electronic Structure of Interfacial Phenol in Water Droplets

Jonas Heitland,[1] Jong Chan Lee,[1] Loren Ban,[1] Grite L. Abma,[1] William G. Fortune,[2]
Helen H. Fielding,[2] Bruce L. Yoder,[1] and Ruth Signorell[1,*]

[1] Department of Chemistry and Applied Biosciences, ETH Zurich, 8093 Zurich, C.H.
[2] Department of Chemistry, University College London, London WC1H 0AJ, U.K.
* rsignorell@ethz.ch

Version of 12 August 2024

S1. POTENTIAL ENERGY CURVES FOR PHOTODISSOCIATION OF AQUEOUS PHENOL

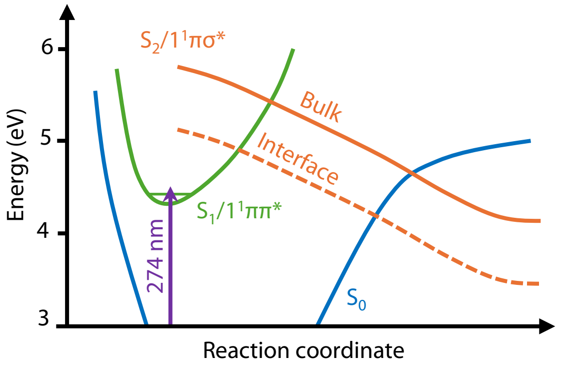

Figure S1. Schematic sketch of the conical intersection between S_1 and S_2 and the potential energy curves along the photodissociation reaction coordinate. The potential energy curve of the S_2 state in bulk aqueous solution (orange, solid) and the lowered curve at the air–water interface (orange, dashed) are shown. The relative potential of the S_1 minimum and the S_1/S_2 conical intersections (CIs) are quantitative quantum chemical calculation results from Ishiyama et al.[1] Note that experiments suggest that at the air–water interface, the CI might already be accessible at 4.64 eV.[2]

S2. EXPERIMENT

S2.1 UV-vis spectrum and laser bandwidth

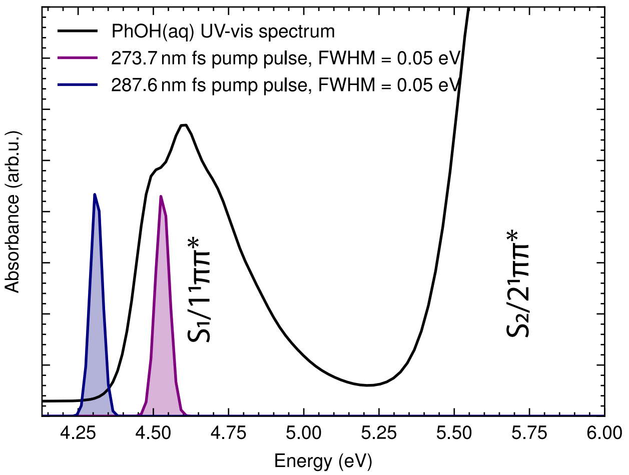

Figure S2. UV-vis absorption spectrum of phenol in aqueous solution. The spectrum is characterized by two bands corresponding to transitions from the S_0 ground state to the $S_1/1^1\pi\pi^*$ and $S_3/2^1\pi\pi^*$ excited states, respectively. The spectral profile of the 274 nm and 288 nm fs pump pulses employed in this work are shown in purple and blue, respectively.

S2.2 Experimental setup

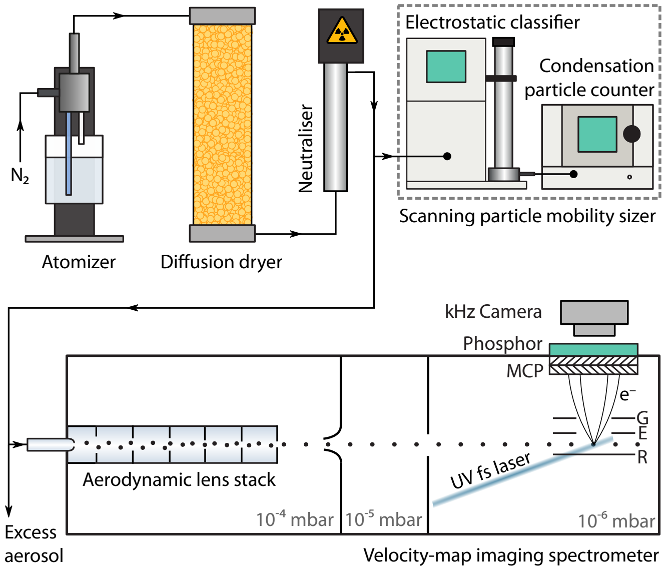

Figure S3. Sketch of the experimental setup consisting of the aerosol droplet generation and conditioning unit (atomizer, silica-based diffusion dryer, neutralizer) and photoelectron spectrometer. The droplets were collimated into a droplet beam and transferred to vacuum via the aerodynamic lens stack (ADL) and ionized by the femtosecond UV laser in between the repeller (R) and extractor (E) plates. Photoelectrons were detected in a three-plate velocity-map imaging configuration [R, E and ground (G) plates] by the imaging detector [microchannel plate (MCP), phosphor screen, camera].

S3. MODELLING

Table S1. Complex refractive indices, $N = n + ik$, of aqueous phenol solution employed in this work for different wavelengths and concentrations of phenol. We assumed n to be equal to that of water in the UV[3,4] and calculated k from the molar extinction coefficient of aqueous phenol solution (S2).[5]

c/M	0.01	0.8
λ/nm		
287.6	$1.376 + i6.228 \times 10^{-7}$	$1.376 + i4.982 \times 10^{-5}$
273.7	$1.382 + i2.716 \times 10^{-5}$	$1.382 + i2.172 \times 10^{-3}$

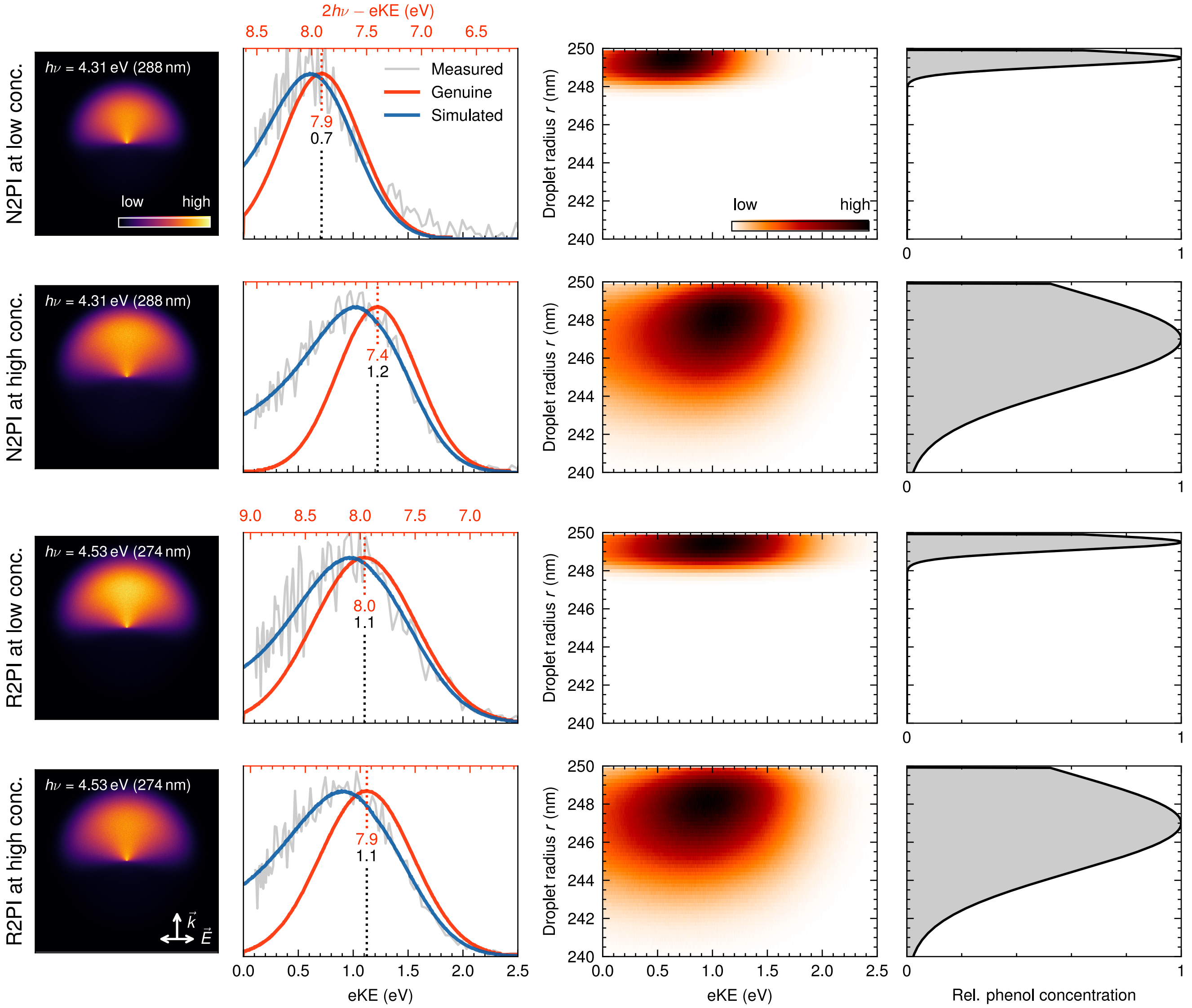

Figure S4. Simulated photoelectron VMIs (first column) and corresponding simulated and measured photoelectron spectra (second column) recorded after two-photon ionization (2PI) of aqueous phenol droplets with a radius of 250 nm. The simulations were performed with our electron scattering program.[6] The arrows in the first column indicate the laser propagation direction $\vec{k}$ and the laser polarization direction $\vec{E}$. The displayed eKE distributions in the second column correspond to the measured (gray), the simulated (blue), and the genuine (red) droplet photoelectron spectra. The centers of the Gaussian-shaped genuine spectra are marked by the vertical dotted lines. A secondary abscissa in red at the top shows the corresponding two-photon electron binding energies (eBE = $2h\nu$ − eKE). Top row: Nonresonant 2PI (N2PI) at 288 nm at low (0.01 M) phenol concentrations. Second row: N2PI at 288 nm at high (0.8 M) phenol concentrations. Third row: Resonance-enhanced 2PI (R2PI) at 274 nm at low (0.01 M) phenol concentrations. Bottom row: R2PI at 274 nm at high (0.8 M) phenol concentrations. The origins of the detected photoelectron as a function of the radial distance r from the droplet's center and the eKE are plotted as a heat map (third column; black: high electron yield, yellow: low electron yield, white: zero electron yield). The phenol surface layer for low and high concentration was modeled by Gaussian-shaped radial concentration profiles of 1 and 6 nm FWHM, respectively (fourth column) in accordance with neutron reflectivity experiments and recent MD simulations.[1,7,8]

S4. DETECTION OF AQUEOUS PHENOL DROPLETS AND IN SITU DETERMINATION OF DROPLET SIZE

S4.1 Three-photon ionization of pure water droplets

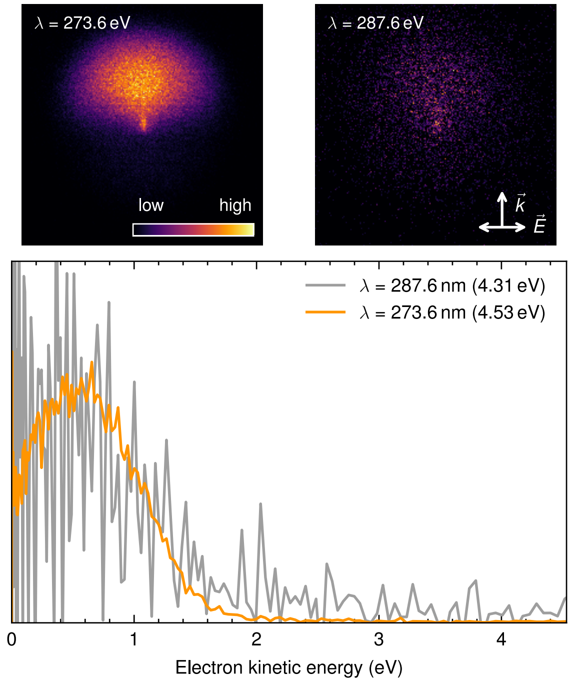

Figure S5. VMIs (top) and photoelectron spectra (bottom) of pure water droplets following three-photon ionization using 274 nm (left VMI; orange spectrum) and 288 nm (right VMI, gray spectrum) light. To record these spectra of pure water, the laser powers were substantially increased compared with the power used for the spectra of the aqueous phenol droplets. We note here that the water background spectra that were subtracted from the aqueous phenol droplet spectra have even lower signal-to-noise than the gray 288 nm water spectrum shown in this figure because of the lower laser powers.

S4.2 TOF spectra of water droplets

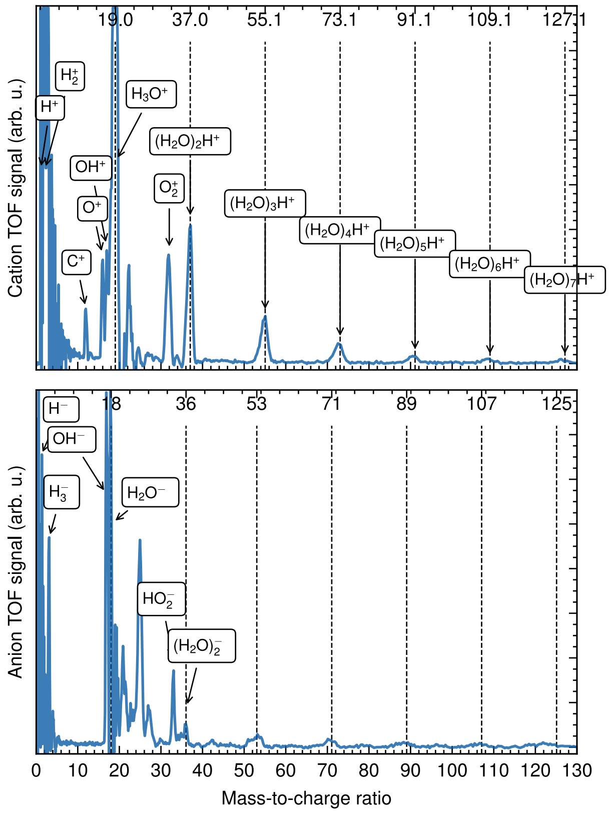

Figure S6. Cation (top) and anion (bottom) time-of-flight mass spectra of the ions ejected after disintegrating/ablating water droplets with intense, focused 800-nm femtosecond laser pulses. The TOF spectra show water clusters up to $n = 8$. Such clusters are detectable only when water droplets are present in the ionization region.

S4.3 In situ size determination

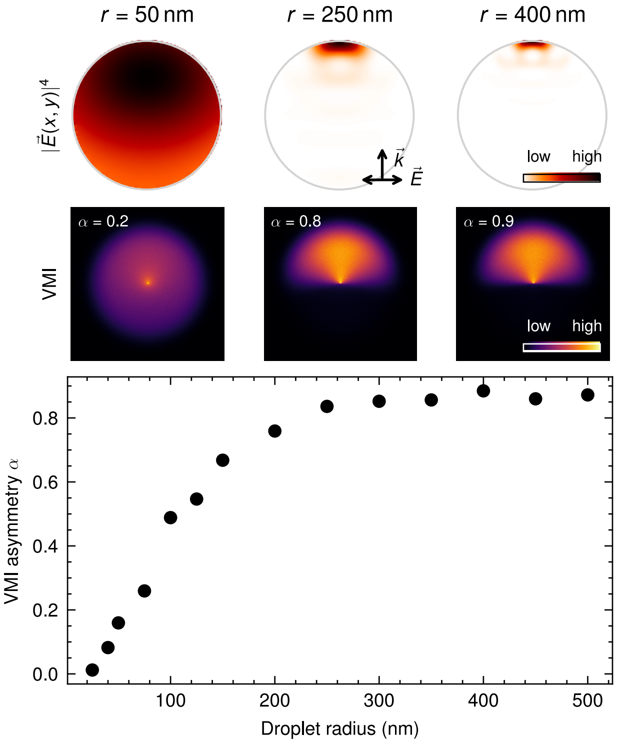

Figure S7. Top row: Center-cuts of the simulated, squared light intensity distribution $|\vec{E}(x, y)|^4$ within aqueous phenol droplets from ADDA calculations. The cuts show strong nanofocusing for the larger droplets. The droplets were irradiated by 274 nm plane-wave light ($N = 1.382 + i2.172 \times 10^{-3}$). The arrows indicate the laser propagation $\vec{k}$ and polarization direction $\vec{E}$. Middle row: Corresponding simulated photoelectron VMIs for 274 nm two-photon ionization of aqueous phenol droplets. The phenol surface layer was modeled by a Gaussian radial concentration profile with a FWHM of 6 nm (Fig. S4). Bottom row: Forward–backward (with respect to $\vec{k}$) asymmetry parameter α of the simulated photoelectron VMIs as a function of droplet radius (Eq. 2, main manuscript). The simulated α values agree well with the measured asymmetry of $\alpha \sim 0.8$ for droplet radii of ~250 nm.

S5. EFFECT OF ELECTRIC CHARGES AND ADDED ALKALI HALIDES ON THE PE SPECTRA

S5.1 Effect of neutralizer

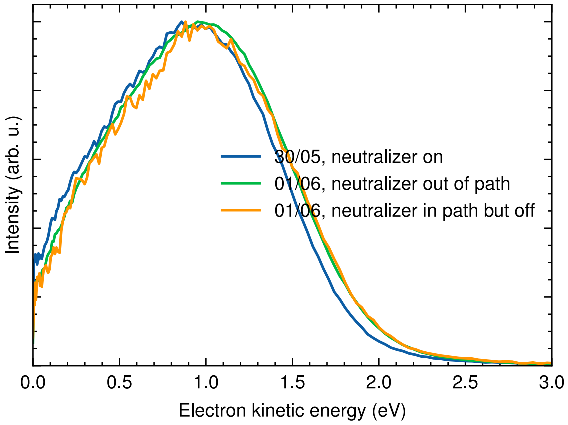

Figure S8. Effect of a soft X-ray bipolar diffusion charger ("neutralizer"): Photoelectron spectra (smoothed) following resonance-enhanced two-photon ionization at 274 nm of 0.1 M PhOH(aq) in water droplets with an aerosol neutralizer in path turned on (blue) vs off (orange). Imposing a net neutral charge distribution on the aqueous phenol particles has only a very minor effect on the photoelectron spectrum. The effect is too small to explain the difference between R2PI droplet and LJ spectra.

S5.2 Effect of added alkali halides on the PE spectra

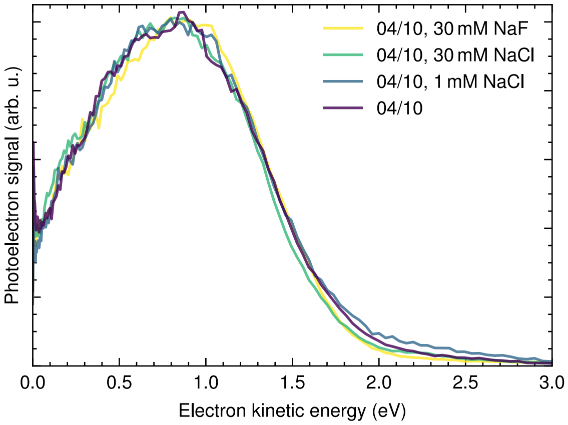

Figure S9. Effect of added alkali halides: Photoelectron spectra (smoothed) following resonance-enhanced two-photon ionization at 274 nm of 0.1 M PhOH(aq) in water droplets with varying amounts of added alkali halides. The alkali halides have no significant effect on the photoelectron spectrum.

REFERENCES

(1) Ishiyama, T.; Tahara, T.; Morita, A. Why the Photochemical Reaction of Phenol Becomes Ultrafast at the Air-Water Interface: The Effect of Surface Hydration. *Journal of the American Chemical Society* **2022**, *144*, 6321–6325.

(2) Kusaka, R.; Nihonyanagi, S.; Tahara, T. The photochemical reaction of phenol becomes ultrafast at the air–water interface. *Nature Chemistry* **2021**, *13*, 306–311.

(3) Querry, M. R.; Hale, G. M. Optical Constants of Water in the 200-nm to 200-μm Wavelength Region. *Applied Optics* **1973**, *12*, 555–563.

(4) Segelstein, D. J. The complex refractive index of water. **1981**, 1–167.

(5) Dearden, J. C.; Forbes, W. F. Light Absorption Studies Part XIV. The Ultraviolet Absorption Spectra of Phenols. *Canadian Journal of Chemistry* **1959**, *37*, 1294–1304.

(6) Luckhaus, D.; Yamamoto, Y.; Suzuki, T.; Signorell, R. Genuine binding energy of the hydrated electron. *Science Advances* **2017**, *3*, e160322.

(7) Li, Z. X.; Thomas, R. K.; Rennie, A. R.; Penfold, J. Neutron reflection study of phenol adsorbed at the surface of its aqueous solutions: An unusual adsorbed layer. *Journal of Physical Chemistry B* **1998**, *102*, 185–192.

(8) Scholz, M. S.; Fortune, W. G.; Tau, O.; Fielding, H. H. Accurate Vertical Ionization Energy of Water and Retrieval of True Ultraviolet Photoelectron Spectra of Aqueous Solutions. *The Journal of Physical Chemistry Letters* **2022**, *13*, 6889–6895.
